# Posttranslational arginylation enzyme Ate1 affects DNA mutagenesis by regulating stress response

**DOI:** 10.1038/cddis.2016.284

**Published:** 2016-09-29

**Authors:** Akhilesh Kumar, Michael D Birnbaum, Devang M Patel, William M Morgan, Jayanti Singh, Antoni Barrientos, Fangliang Zhang

**Affiliations:** 1 Department of Molecular and Cellular Pharmacology, University of Miami Leonard M. Miller School of Medicine, Miami, FL 33136, USA; 2 Department of Medicine, Division of Cardiology, University of Miami Leonard M. Miller School of Medicine, Miami, FL 33136, USA; 3 Department of Neurology, University of Miami Leonard M. Miller School of Medicine, Miami, FL 33136, USA; 4 Department of Biochemistry and Molecular Biology, University of Miami Leonard M. Miller School of Medicine, Miami, FL 33136, USA; 5 Sylvester Comprehensive Cancer Center, University of Miami Leonard M. Miller School of Medicine, Miami, FL 33136, USA

## Abstract

Arginyltransferase 1 (Ate1) mediates protein arginylation, a poorly understood protein posttranslational modification (PTM) in eukaryotic cells. Previous evidence suggest a potential involvement of arginylation in stress response and this PTM was traditionally considered anti-apoptotic based on the studies of individual substrates. However, here we found that arginylation promotes cell death and/or growth arrest, depending on the nature and intensity of the stressing factor. Specifically, in yeast, mouse and human cells, deletion or downregulation of the *ATE1* gene disrupts typical stress responses by bypassing growth arrest and suppressing cell death events in the presence of disease-related stressing factors, including oxidative, heat, and osmotic stresses, as well as the exposure to heavy metals or radiation. Conversely, in wild-type cells responding to stress, there is an increase of cellular Ate1 protein level and arginylation activity. Furthermore, the increase of Ate1 protein directly promotes cell death in a manner dependent on its arginylation activity. Finally, we found Ate1 to be required to suppress mutation frequency in yeast and mammalian cells during DNA-damaging conditions such as ultraviolet irradiation. Our study clarifies the role of Ate1/arginylation in stress response and provides a new mechanism to explain the link between Ate1 and a variety of diseases including cancer. This is also the first example that the modulation of the global level of a PTM is capable of affecting DNA mutagenesis.

As a common response to changing environmental cues, posttranslational modifications (PTMs) of proteins can promptly modulate cellular functions without *de novo* translation or transcription. Among them, arginylation is the posttranslational addition of an extra arginine to an existing peptide chain, usually to the N terminus, thus capable of changing the surface charge as well as the primary sequence of the target.^1^ Arginylation is mediated by arginyltransferase 1 (Ate1),^2, 3^ an evolutionarily conserved enzyme found in eukaryotic organisms and some bacteria.^4, 5, 6^ Nearly a hundred proteins have been found to be arginylated and the list of identified substrates is continuously growing,^7, 8^ suggesting a widespread effect of this PTM *in vivo*.

Mounting evidence has suggested an involvement of arginylation in stress response, a naturally occurring process undertaken by cells in stressing conditions, which often lead to growth arrest or cell death. For more than two decades, researchers have repeatedly observed signs of increased arginylation activity in tissues of animals (such as rat, chicken and fish) following a variety of insults including nerve crush injury or whole-body hyperthermia.^9, 10, 11, 12, 13, 14, 15, 16, 17, 18^ At the molecular level, arginylation is often observed on proteins that are nitrosylated, oxidized or misfolded, which are common consequences of stress.^19, 20, 21^ However, the exact nature of arginylation's involvement in these processes remains unclear. According to the N-end rule, a protein degradation theory that correlates the metabolic stabilities of a peptide to the identity of its N-terminal residue, arginylation constitutes an acute degradation signal conferring a half-life of <3 min.^22^ For this reason, previous studies mainly focused on identifying novel substrates for cues of mechanistic insights of arginylation, under the assumption that this modification should lead to a quick degradation. On this basis, several studies suggested that a normal activity of arginylation is required for anti-apoptotic activities through proteins such as drosophila inhibitor of apoptosis protein, and degradation of pro-apoptotic fragments of RIPK1 (receptor-interacting serine/threonine protein kinase 1) and BRCA1 (breast cancer 1, early onset) created by the action of caspase or calpain.^23, 24, 25, 26, 27^ Therefore, Ate1-mediated arginylation has been traditionally considered to have an anti-apoptotic effect *in vivo*.^23, 27, 28, 29^ However, arginylation affects a broad range of proteins with diverse functions, some of which are arguably anti-apoptotic.^30^ Further complicating the matter, arginylation may regulate protein structure and function independently of protein half-life.^31, 32, 33, 34, 35^ Hence, it may be ineffective or inaccurate to ascribe a global function to Ate1 based on the identities of a few individual substrates. A new approach is needed to directly evaluate the overall molecular impact of Ate1-mediated arginylation regardless of individual substrates.

To test the role of Ate1 and arginylation in stress response, we utilized multiple eukaryotic systems including *Saccharomyces cerevisiae* (budding yeast), mouse cells and human cells, all of which contain the evolutionarily conserved *ATE1* gene. We found that, when arginylation activity is globally downregulated by the deletion or silencing of the *ATE1* gene, cellular sensitivities to a variety of stressing factors were significantly reduced, resulting in bypass of growth arrest or reduction of cell death under stress. In addition, the Ate1 protein level and the global arginylation activity are increased in cells under stress, and Ate1 mediates cell death with an arginylation-dependent manner. Finally, Ate1 is needed for suppressing the outcome of DNA mutagenesis during DNA-damaging stress. To our knowledge, our finding is the first example of a PTM having a global effect on DNA mutagenesis.

## Results

### Removal or downregulation of Ate1 disrupts stress response and reduces cellular sensitivity to a variety of stressing conditions

**Figure 1:**
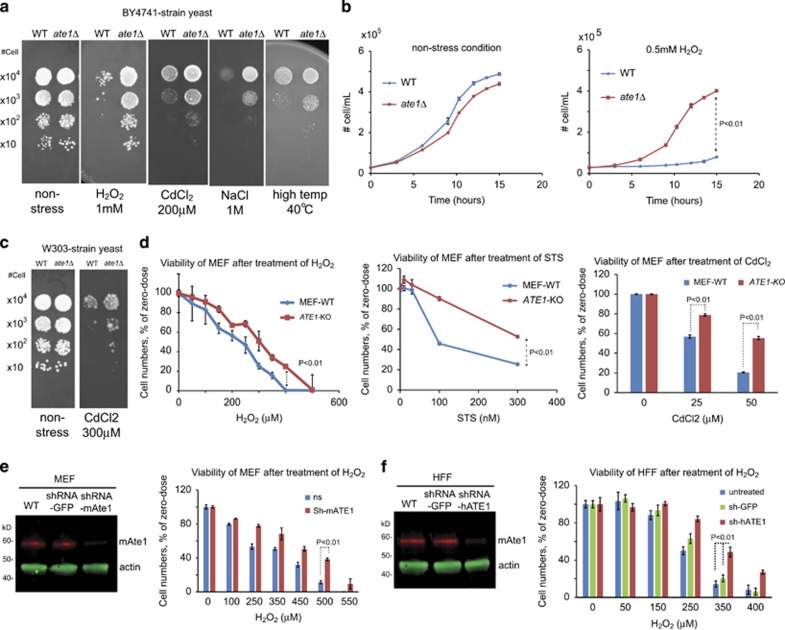
Knockout or knockdown of *ATE1* decreases cellular sensitivity towards stressing conditions. (**a**) Growth test using serial dilutions of wild-type (WT) and *ate1*Δ yeast (*S. cerevisae*, BY4741 strain, unless otherwise mentioned) in non-stressing condition or in the presence of one of the following stressors: common cellular oxidant (H_2_O_2_, 1 mM), heavy metal (CdCl_2_, 200 *μ*M), high salt (NaCl, 1 M) or high temperature (40 °C). For H_2_O_2_ treatment, cells were plated on SD plates with glucose. For the other treatments and the non-stressing controls, cells were plated on YPD plates. (**b**) Growth curves of WT and *ate1*Δ yeast cultured in liquid media, in the presence or absence of the oxidative stressor H_2_O_2_. Error bars represent S.D., *n*=3. (**c**) Growth test using WT or *ate1*Δ yeast (strain W303) in non-stressed conditions or in the presence of the heavy metal CdCl_2_ (300 *μ*M). (**d**) Viabilities of WT or *ATE1*-KO mouse embryonic fibroblasts (MEFs) after 12 h treatments with increasing concentrations of cellular oxidant H_2_O_2_, bacterial toxin STS or heavy metal CdCl_2_. The number of viable cells after H_2_O_2_ treatment was measured by Calcein AM, a cellular dye that emits fluorescence only in live cells. The number of viable cells after STS and CdCl_2_ treatments was directly counted with an automated cell counter (TC-20 from Bio-Rad) with the cross-staining of Trypan blue, which stains dead cell but not live cells. In each type of cell, one sample of cells with control treatment (DMSO only) was used as normalization for other samples, for WT or *ATE1*-KO, respectively. Error bar represents S.E.M. of multiple repeats (*n*=4 for H_2_O_2_ and STS, and *n*=3 for Cdcl_2_). (**e**) Immunoblot analysis of the steady-state levels of mammalian Ate1 (mAte1) in MEF transfected with either shRNA specific against mouse *ATE1* (sh-m*ATE1*) or shRNA against GFP (used as a non-targeting control). The band of mAte1 was probed with a monoclonal antibody (Millipore, clone #6F11) recognizing all four major splicing variants of Ate1 in mammalian cells, which are almost identical in molecular sizes and poorly understood in functional differences. Actin was used as a loading control. The right panel graph shows the quantification of viability of MEFs transfected with Ate1 knockdown or non-targeting control, after the treatment with H_2_O_2_ for 12 h, as measured by the cellular viability indicator Calcein AM. Error bars represent S.E.M. (*n*=4). (**f**) Similar to **e**, except that human foreskin fibroblast (HFF) and shRNA against human ATE1 (sh-hATE1) were used. Error bars represent SEM (*n*=4). Throughout this study, the *P*-value was calculated by two-tailed Student's *t*-test between specific data points, unless otherwise indicated

Ate1 is coded by a single gene in yeast and mammals.^4, 5^ When we deleted the evolutionarily conserved *ATE1* gene in the budding yeast, *S. cerevisiae* (strain BY4741, unless otherwise indicated), we found no obvious effect on growth in non-stressing conditions in nutrient-rich medium (Figures 1a and b). Upon exposure to stress, including H_2_O_2_-induced oxidative stress, heavy metals, high salt or high temperature, wild-type (WT) yeast grew at a significantly lower rate compared with non-stressing conditions, which is an expected outcome of normal stress response (Figures 1a and b). However, the growth of *ate1*Δ yeast was less affected by these stressors (Figures 1a and b), suggesting a disruption of stress response.^36^ The deletion of the *ATE1* gene in a different yeast strain, W303, similarly increased cellular resistance to CdCl_2_ (Figure 1c).

To test whether Ate1 has a similar role in mammals, we compared WT and *ATE1-*KO mouse embryonic fibroblasts (MEFs) and found that deletion of the *ATE1* gene increased cellular resistance to stressors such as cellular oxidant H_2_O_2_, heavy metal CdCl_2_ and microbial alkaloid toxin staurosporine (STS; Figure 1d). In addition, when *ATE1* expression was attenuated by short hairpin RNA (shRNA)-mediated knockdown in MEFs and human foreskin fibroblasts (HFFs), resistance to the oxidant H_2_O_2_ was increased (Figures 1e and f).

**Figure 2:**
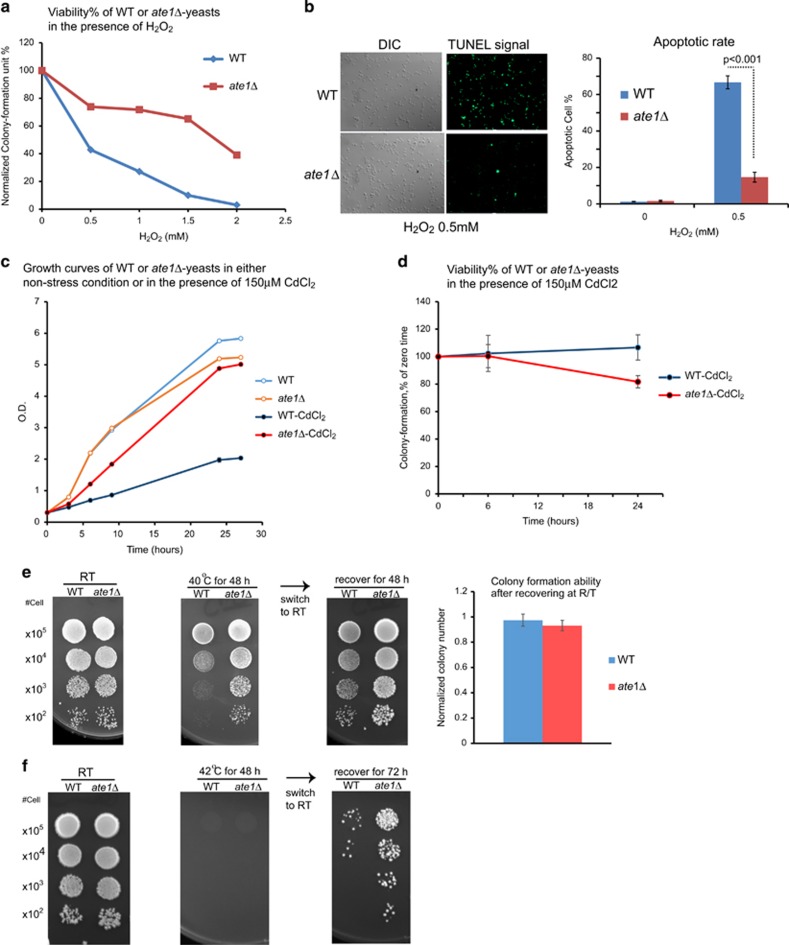
Knockout of ATE1 in yeast relieves growth arrest and suppresses cell death during stress response. (**a**) Viability of wild-type (WT) or *ate1*Δ yeast after 3 h treatments with the indicated concentrations of the oxidative stressor H_2_O_2_, measured by the colony-forming unit assay and normalized to non-stressed conditions. (**b**) On the left are representative microscopic images showing the results of the TUNEL assay, which specifically detects DNA fragments generated during late stage of apoptosis. The WT and *ate1*Δ yeast treated with H_2_O_2_ 0.5 mM. DIC images show the number of cells while the fluorescent images show cells with positive apoptosis signals (fluorescein). The graph in the right panel represents the percentage of apoptotic cells determined by TUNEL assay in WT and *ate1*Δ yeast treated with increased concentrations of H_2_O_2_. (**c**) Growth curves of WT and *ate1*Δ yeast cultured in liquid media, in the presence or absence of the heavy metal stressor CdCl_2_ of 150 *μ*M. Error bars represent S.E.M. (*n*=3). (**d**) Viability of WT or *ate1*Δ yeast after indicated hours of treatments with 150 *μ*M CdCl_2_, measured by the colony-forming unit assay and normalized to cells at time 0 (before the application of stressor). Error bars represent S.E.M. (*n*=3). (**e**) Growth test using serial dilutions of WT and *ate1*Δ yeast, either in non-stressing room temperature (RT) or in high temperature (40 °C) for 48 h followed by recovery at RT for another 48 h. After recovery at RT, the numbers of colonies emerged in the lowest dilution (× 10^2^) were quantified. The corresponding numbers for yeast constantly cultured at RT was used for normalization for WT and *ate1*Δ yeast separately. Error bars represent S.E.M. (*n*=4 for WT, *n*=3 for *ate1*Δ). (**f**) Growth test using serial dilutions of WT and *ate1*Δ yeast, either in non-stressing RT or in high-temperature (42 °C) for 48 h followed by recovery at RT for another 72 h

Common effects of stress response include cell death and growth arrest. To test whether the deletion of *ATE1* affects cell death in yeast, we examined cellular viabilities by colony-forming per cells optical density (OD) unit (CFU) in yeast cultures in the presence of lethal doses of H_2_O_2_. We found that *ate1*Δ yeast cultures had higher percentages of viable cells compared with the WT (Figure 2a). Furthermore, using the terminal deoxynucleotidyl transferase dUTP nick end labeling (TUNEL) assay to probe apoptosis, a programmed cell death event, we found that the deletion of *ATE1* greatly attenuated H_2_O_2_-induced apoptosis (Figure 2b), which contradicts the prevailing hypothesis for the anti-apoptotic roles for Ate1 and arginylation.^23, 28, 29^

To examine whether Ate1 also has an impact on growth arrest, we exposed yeast cultures to a moderate concentration (150 *μ*M) of CdCl_2_. This treatment led to a much lower growth rate in the WT yeast culture compared with *ate1*Δ (Figure 2c). However, both cultures had similar viabilities as assessed by the CFU assay (Figure 2d). In fact, in the late sampling time point (24 h), the WT yeast even appeared to have slightly higher viability compared with *ate1*Δ, probably due to nutrient constraints in the *ate1*Δ yeast culture because they reached a saturation density at that time point. Therefore, the faster growth rates of *ate1*Δ yeast in stressing condition was likely caused by a lack of growth arrest.

To further test whether Ate1 affects both growth arrest and cell death for the same stressor, we challenged the WT and *ate1*Δ yeast with high-temperature conditions. At 40 °C, a heat-stress temperature for yeast, the *ate1*Δ yeast grew significantly faster than the WT. However, when these yeast cultures were transferred from 40 °C to room temperature (RT), a non-stressing temperature for recovery, similar numbers of colonies eventually formed in both the *ate1*Δ and WT yeast. This suggests that both yeast strains were equally viable, and their difference in growth at 40 °C was mainly due to a difference in growth arrest (Figure 2e). However, when yeast were incubated at 42 °C and then transferred to RT, *ate1*Δ yeast were able to form significantly more colonies than the WT yeast, indicating that a deletion of *ATE1* in this condition yielded a higher cell survival rate (Figure 2f). Therefore, the lack of Ate1 may lead to the bypass of growth arrest and/or the suppression of cell death from the same stressor, dependent on the intensity of stressor.

**Figure 3:**
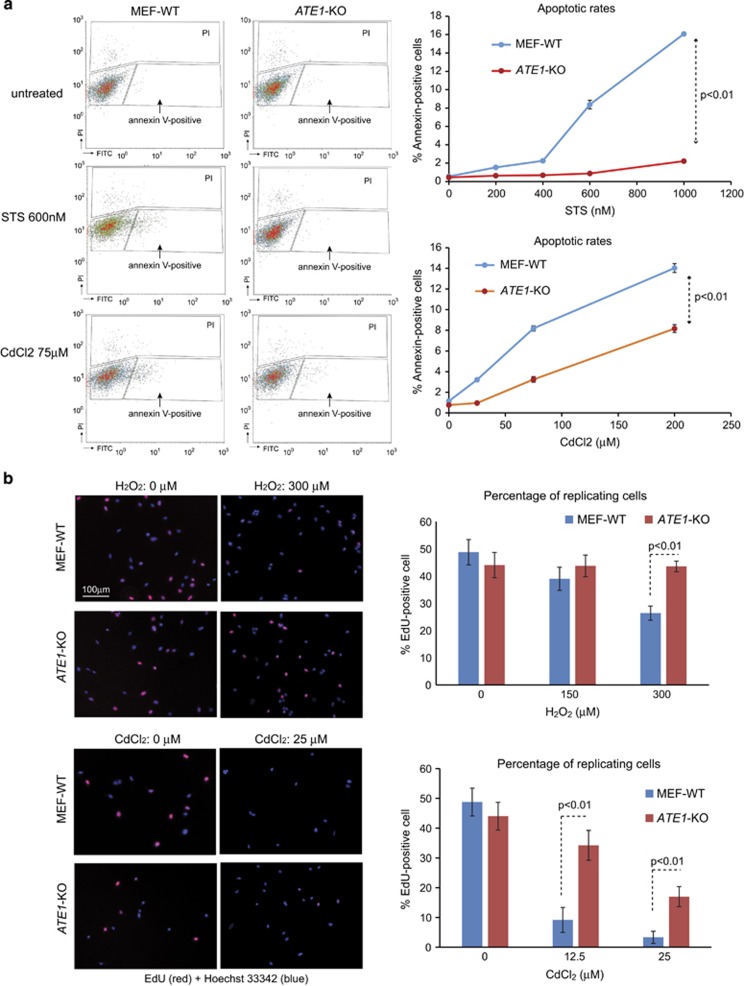
Knockout of ATE1 in MEFs results in attenuated apoptosis and growth arrest during stress response. (**a**) Annexin-V-Alexa Fluor 488 (Thermo Fisher) was used to detect phosphatidylserine inversion on the peripheral membrane in an early stage of apoptosis. WT or *ATE1*-KO MEFs were treated with STS or CdCl_2_ of different concentrations for 5 h. Propidium iodide (PI) was used to label necrotic and late apoptotic fractions. The cell population is analyzed by FACS. On the left panel representative FACS charts are shown. The gate settings for Annexin-V and PI detection are indicated. Quantification of data from three independent repeats (*n*=3) are shown in graphs presented on the right side. *ATE1*-KO cells have a much lower apoptotic rate than WT cells. No obvious difference in PI staining was found in the conditions we tested. (**b**) On the left panel are the representative microscopic images showing WT and *ATE1*-KO MEFs treated with H_2_O_2_ 0.5 mM. EdU (red fluorescently labeled), which is incorporated into newly synthesized DNA during the S phase of cell cycle, was used as a marker for cell proliferation. Hoechst 33342 (blue) was used to show the number and morphology of nucleus. Only cells with normal nuclear morphology and no sign of apoptotic bodies were included for quantification. At least 10 randomly chosen images in each group were used to generate the graphs shown on the right side for quantification of active replicating cells with EdU incorporated into their nucleus, in the presence of stressors H_2_O_2_ and CdCl_2_. Error bars represent S.E.M.

To test whether mammalian Ate1 (mAte1) mediates apoptosis, we challenged WT and *ATE1-*KO MEFs with apoptosis-inducing reagent STS of relatively high doses (200–1000 nM) for a short duration (5 h). By employing the Annexin-V staining assay to detect early apoptotic signals, we found that the knockout (KO) of *ATE1* gene in MEFs significantly reduced apoptotic rates in the presence of STS (Figure 3a). Similar results were observed with a different stressor, CdCl_2_ (Figure 3a). Next, to test whether mAte1 regulates growth arrest in stressing conditions, we challenged the WT and *ATE1-*KO MEFs with either H_2_O_2_ or CdCl_2_, with lower doses and longer durations (12 h). Using a thymidine analog, 5-ethynyl-2′-deoxyuridine (EdU) to measure cell proliferation activity, we found no significant difference between WT and *ATE1-*KO MEFs in non-stressing conditions. However, in the presence of stressors, the percentage of EdU-positive population in WT cells is significantly lower than in *ATE1-KO* MEFs, suggesting that the absence of *ATE1* prevents growth suppression of mammalian cells under stress (Figure 3b).

### Ate1 and arginylation are upregulated during stress and are responsible for cell death

**Figure 4:**
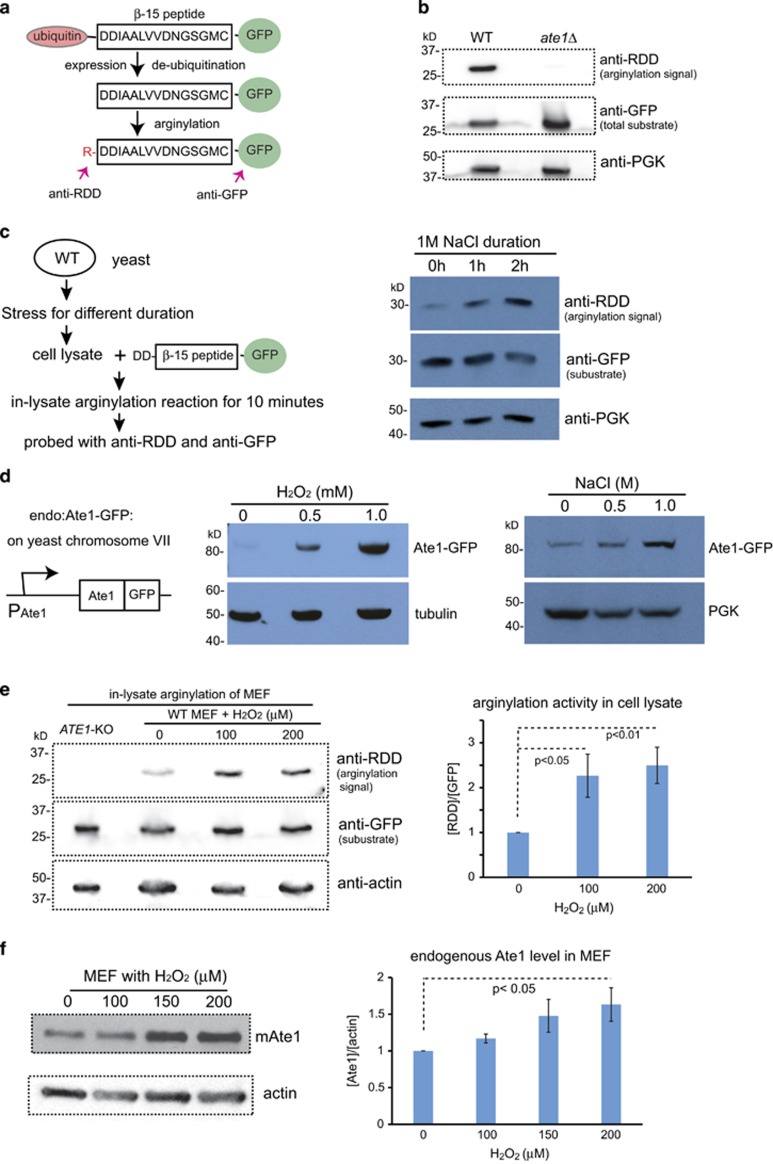
The levels of Ate1 protein and global arginylation activity are upregulated during stress. (**a**) A scheme illustrating how DD-*β*15-GFP is used as the reporter of arginylation activity. The fusion protein containing a stretch of 15 amino acids starting with two aspartic acids (D) derived from the N terminus of mammalian *β*-actin, a known substrate of arginylation.^31^ This peptide is fused with an N-terminal ubiquitin, which is cleaved co-translationally by endogenous de-ubiquitylation enzymes in eukaryotic system and leaves the aspartic acids as the new N terminus. The arginylation state of this reporter can be probed with an anti-RDD antibody, which only reacts with the arginylated form of the reporter protein. A C-terminal GFP tag is used to facilitate the detection of steady-state level of the reporter protein by immunoblotting with anti-GFP antibody. (**b**) The arginylation level of DD-*β*15-GFP expressed in either WT or *ate1*Δ yeast was examined with anti-RDD antibody, which only shows a visible signal in the WT cells. An antibody for GFP was used to probe the total protein level of the DD-*β*15-GFP. PGK was used as a loading control for total yeast cellular proteins. (**c**) Illustrative scheme (left panel) and representative immunoblots (right panel) showing the arginylation activity in cell lysates prepared from yeast exposed to 1 M NaCl stress for increasing times. The lysates were then mixed with the recombinant protein DD-*β*15-GFP prepared from *ate1*Δ yeast for an in-lysate arginylation reaction for 10 min at RT. The arginylation level of the reporter protein was detected by immunoblotting with anti-RDD antibody. The steady-state level of the reporter protein was probed with anti-GFP. An anti-3-phosphoglycerate kinase (PGK) antibody was also used as a loading control. (**d**) On the left, a scheme illustrating the domain structure of the ‘*in locus*' GFP-fused Ate1, which is driven be the endogenous *ATE1* promoter at the native chromosome locus (Chromosome VII) in the yeast (termed ‘endo: Ate1-GFP'). The right panels present immunoblots showing the steady-state levels of ‘endo: Ate1-GFP' in yeast treated with increasing concentrations of different stressors: H_2_O_2_ (left) or NaCl (right). Tubulin or PGK was used as loading controls. (**e**) WT MEFs were exposed to increased concentrations of H_2_O_2_ for 30 h. The lysates from all these cells, as well as untreated *ATE1*-KO MEFs (as a control), were then mixed with the recombinant protein DD-*β*15-GFP purified from *ate1*Δ yeast for an in-lysate arginylation reaction for 45 min at RT. The arginylation level of the reporter protein was detected by immunoblotting with anti-RDD antibody. The steady-state level of the reporter protein was probed with anti-GFP. Actin antibody was used as a loading control. The graph on the right side shows quantification from four independent repeats. (**f**) Left: representative immunoblots showing the levels of endogenous Ate1 proteins in MEFs treated with increasing concentrations of H_2_O_2_ for 30 h, detected by a specific antibody for mouse Ate1 (Millipore, clone 6F11) recognizing all four major Ate1-splicing variants. Actin was used as loading controls. Right: quantification of the endogenous Ate1 level in MEFs treated with H_2_O_2_ from three independent repeats. The Ate1 level was calculated by normalization with actin loading control, and then further normalized to the level at non-stressing condition (0 *μ*M H_2_O_2_). In all above figures, error bars represent S.E.M. and statistical significance was calculated by Student's *t*-test

In yeast and mammals, only one *ATE1* gene was identified.^4, 5^ To confirm that the deletion of this gene can abolish cellular arginylation activity, we designed a reporter substrate termed DD-*β*15-GFP (Figure 4a). When this reporter protein was expressed in yeast cells, we found that its arginylated form existed only in WT yeast, but not in *ate1*Δ (Figure 4b). A similar observation was made in mammalian MEFs (Figure 4e). As a minor note, the steady-state level of the reporter protein, as probed by anti-GFP, was obviously lower in WT yeast than in *ate1*Δ, likely due to an arginylation-mediated degradation in yeast (Figure 4b).

In most past studies, arginylation was demonstrated by radioactively labeled arginine, which can be incorporated into protein by arginylation or translation. To separate these two effects, translation was inhibited by either inhibitors or depletion of ribosomes.^9, 10, 11, 12, 13, 14^ However, most translation inhibitors are unfortunately leaky, and removal of ribosomes can collaterally deplete Ate1 due to their known affinity.^37^ To remove these ambiguities, we used DD-*β*15-GFP for an ‘in-lysate' reaction to examine arginylation activity in cell extracts (Figure 4c, left panel). Using this assay, we found an increase of arginylation signal in WT yeast exposed to NaCl as an osmotic stressor, in proportion to stress durations (Figure 4c, right panel). Also, the level of the substrate is reduced proportionally, likely due to arginylation-mediated degradation (Figure 4c, right panel and middle strip). As a control, extracts from *ate1*Δ yeast exposed to the same treatment did not show an increase (or any signal) of the arginylated reporter or a reduction of the substrates (Supplementary Figure S1), suggesting that the stress-induced increase of arginylation depends on the presence of Ate1. Consistent with the observation in yeast, a dose-dependent increase of arginylation activity was found in MEFs treated with H_2_O_2_ (Figure 4e). These data indicate that the global arginylation activity in the cell is upregulated as part of the natural stress response process, which is consistent with previous observations.^9, 10, 11, 12, 13, 14^

To test whether Ate1 protein is also upregulated in stress response, we took advantage of a commercially available yeast strain carrying an ‘*in locus*' 3′-end fusion of green fluorescent protein (GFP) with the endogenous *ATE1* gene (termed ‘endo: Ate1-GFP') under the control of the endogenous *ATE1* promoter. We found that the protein level of ‘endo: Ate1-GFP' was proportionally increased with the dose of each stressors such as H_2_O_2_ or high salt, (Figure 4d). Similarly, when we probed the steady-state level of endogenous Ate1 in MEFs, we found a dose-dependent increase of total Ate1 protein in the presence of H_2_O_2_ (Figure 4f). These data suggest that Ate1 protein level is upregulated during stress in yeast and mammalian cells.

**Figure 5:**
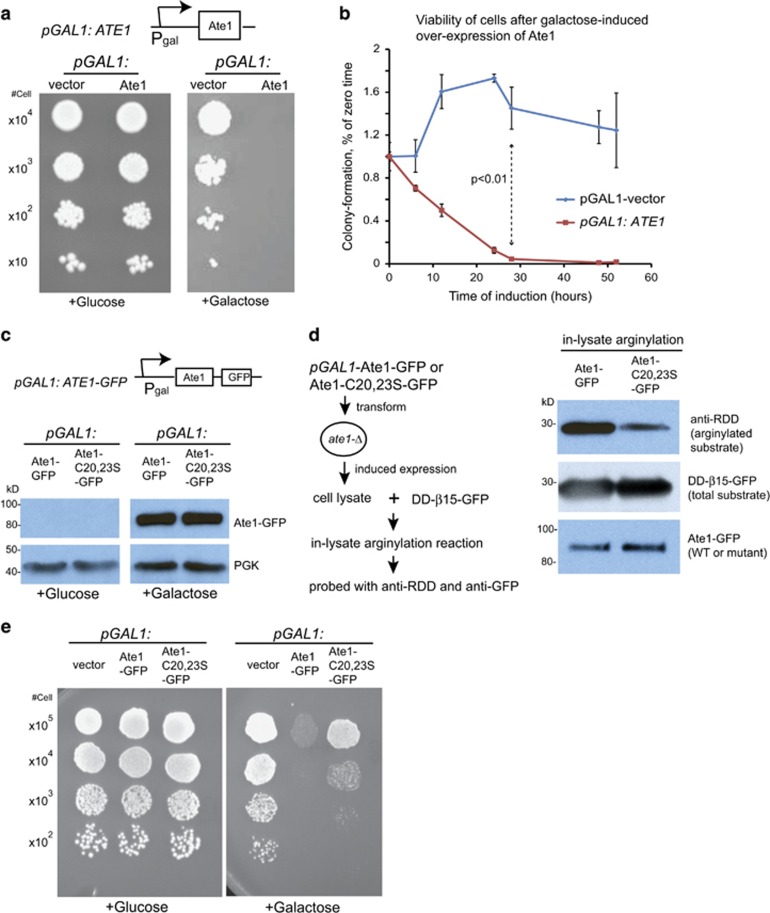
The increase of Ate1 triggers cell death in yeast in a manner that is dependent on its arginylation activity. (**a**) The scheme in the top panel shows the domain structure of plasmid *pGAL1: ATE1*, in which the coding sequence of recombinant protein is preceded by the inducible *GAL1* promoter. The picture in the bottom panel shows the growth of *ate1*Δ yeast cells carrying either the empty expression vector or *pGAL1: ATE1* by a serial dilution growth assay on either plate containing glucose (suppressing) or galactose (inducing). (**b**) Graph showing the viabilities of *ate1*Δ yeast cells carrying either the empty expression vector or *pGAL1: ATE1* in different time points following the initiation of galactose-induced expression, as measured by the numbers of colony-forming cells per OD unit (CFU) that were normalized to starting data point time 0, for Ate1 or vector control separately. Error bar represents S.E.M. (*n*⩾3). (**c**) The top scheme shows the domain structure of a recombinant Ate1 fused with a linker and a C-terminal GFP, driven by the pGAL1 promoter, termed ‘*pGAL*: Ate1-GFP'. Immunoblot analysis of the steady-state levels of wild-type and C20,23S mutant Ate1 after 9-h culture in the presence of non-inducing (glucose) or inducing (galactose) carbon sources. PGK was used as loading controls. Anti-GFP was used to probe the steady-state levels of the recombinant ‘*pGAL*: Ate1-GFP' (WT or mutant). (**d**) Left panel showing the procedure of using an in-lysate arginylation assay to measure the activities of recombinant Ate1-GFP, either the WT version or the C20,23S mutant, which were expressed for 9 h in *ate1*Δ yeast (see **c** for the steady-state levels of expressed proteins). Anti-RDD was used to indicate the level of arginylated reporter. Anti-GFP was used to show the total amount of reporter protein (DD-*β*15-GFP) in each sample, as well as the total amount of Ate1-GFP (either WT or mutant) present in each sample. These two bands were distinguished by their difference in molecular weight (27 kDa *versus* 92 kDa). (**e**) Representative pictures of growth test using serial dilutions of *ate1*Δ yeast carrying either the empty expression vector or *pGAL1*-Ate1-GFP, or *pGAL1*-Ate1-C20,23S-GFP, in media containing non-inducing (glucose) or inducing (galactose) carbon sources

As cell death is a common outcome of extended stress response,^36^ we wanted to know whether the upregulation of Ate1/arginylation could be a direct inducer of cell death. To specifically test the effects of Ate1 on cell death, we employed a galactose-inducible recombinant Ate1 (termed ‘*pGAL1:*Ate1') plasmid in yeast. To avoid interference of the endogenous Ate1, we transformed *ate1*Δ yeast with this plasmid (or an empty vector as control). We found that the growth of yeast carrying the inducible Ate1 is strongly repressed on galactose-containing plate, where the expression of the recombinant protein is turned on (Figure 5a). A similar effect was observed with the induced expression of a GFP-fused Ate1 (*pGAL*: *ATE1*-*GFP)* (Figure 5e). To verify whether the increased abundance of Ate1 can induce cell death, yeast cells were induced in galactose-containing liquid medium for increasing durations. The cell viability was then measured by a CFU assay in glucose-containing agar plates (where the galactose induction is terminated). We found that the viability of cells carrying the *pGAL*: *ATE1*-*GFP* was markedly decreased along the time line of galactose induction, suggesting that the upregulation of Ate1 is indeed capable of inducing cell death in yeast (Figure 5b).

To further correlate Ate1 effects on stress response with its arginylation activity, we replaced two critical cysteine (C) residues (20 and 23) with serine (S) on Ate1. These mutations were reported to minimize the enzymatic activity of Ate1 without affecting its solubility, based on *in vitro* studies.^38^ Consistently, we found that the expression level of the C20,23S mutant Ate1 was similar to the WT version of Ate1, suggesting that these mutations did not affect the overall synthesis and turnover of this protein (Figure 5c). To verify whether such mutations indeed reduce Ate1 activity,^37^ we used the in-lysate arginylation assay with DD-*β*15-GFP reporter and found that the activity of the mutation is <25% of the WT enzyme (Figure 5d). When we compared the cells overexpressing the mutant Ate1 and WT Ate1, we found that the C20,23S mutation can significantly reverse the repressing effects of Ate1 overexpression in cells (Figure 5e). Therefore, the effects of yeast Ate1 on stress response is largely (if not completely) dependent on its arginylation activity.

**Figure 6:**
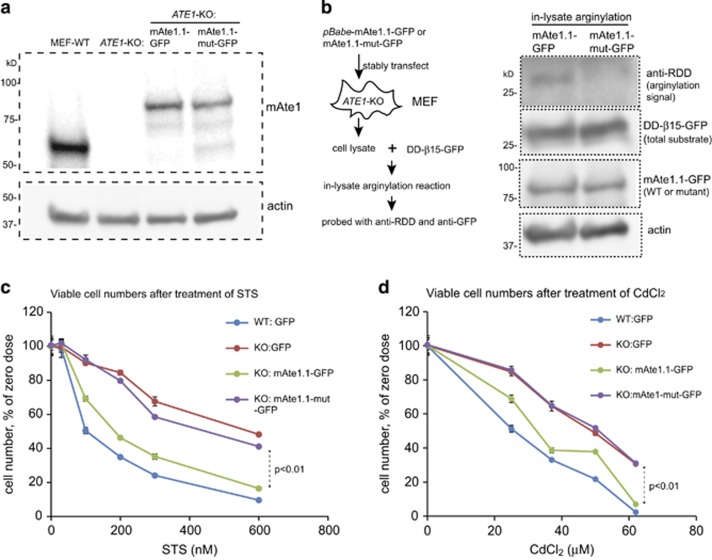
Mammalian Ate1 is required for cellular sensitivity to stressors in a manner dependent on its arginylation activity. (**a**) Representative immunoblots showing the levels of Ate1 protein detected by a specific mouse Ate1 antibody (Millipore, clone 6F11) as either endogenous Ate1 in WT MEF or the recombinant mouse Ate1-isofrom 1 (WT or C23-26S mutant) fused with a C-terminal GFP stably transfected in *ATE1*-KO cells. Actin was used as a loading control. (**b**) Left panel showing the procedure of using an in-lysate arginylation assay to measure the activities of either the WT version of mAte1.1-GFP or the mutant with cysteine 23 and 26 to serine replacement (referred as mAte1.1-mut-GFP in this study) expressed in stably transformed *ATE1*-*KO* MEF. The arginylation reporter protein, DD-*β*15-GFP, as described in Figure 4, was expressed and purified from *ate1*Δ yeast. On the right panel, anti-RDD was used to probe the level of arginylation on the reporter protein. Anti-GFP was used to show the total amount of reporter protein (DD-*β*15-GFP) added in each sample. Mammalian Ate1 antibody (Millipore, clone# 6F11) was used to detect the level of mAte1.1-GFP (either WT or mutant) present in each sample. Actin was used as a loading control for cell lysates. (**c**) Graph showing the quantification of cell viability after treatment of different concentrations of STS for 12 h, for WT and *ATE1*-KO cells stably expressing either GFP (as transfection and expression control), GFP-fused recombinant mouse Ate1.1 or enzymatically impaired mutant mouse Ate1.1. The viable cells were counted by cell counter and using Trypan blue to exclude dead cells. For each group, the data were normalized to a sample under non-stressing condition. Error bars represent S.E.M. (*n*=3). (**d**) Similar to (**c**), except that a treatment of CdCl_2_ was used. Error bars represent S.E.M. (*n*=3)

To test whether mAte1 has similar effects, we used splice variant 1 of mouse Ate1 (referred to as mAte1.1), the most ubiquitous isoform that exists in all tissues of animals,^39^ as the representative. The C-terminal GFP-fused recombinant protein was reintroduced into *ATE1*-KO MEFs by a stable low-expression system that generates recombinant proteins at a level comparable to the endogenous level of WT Ate1 (Figure 6a).^40^ We found that the expression of mAte1.1 was able to reinstall cellular sensitivity to stressors, such as STS and CdCl_2_ (Figures 6c and d), to levels close to WT cells, indicating that mAte1 is indeed mediating cell death in stress response. mAte1 also carries two cysteine residues (C23 and C26 in mAte1) corresponding to the C20 and C23 residues in yeast Ate1. Consistent with our observations in yeast Ate1, when these two cysteine residues were changed to serine in mouse Ate1, the resulted mutant (mAte1.1-mut) had compromised arginylation activity (Figure 6b), and had significantly lower capacity to reinstall cellular sensitivity to STS or CdCl_2_ than the original mAte1.1 (Figures 6c and d). These data indicate that the arginylation activity of mAte1 is also required for its action in mediating stress response.

### Ate1 is essential for the suppression of mutagenesis during DNA-damaging stress

Growth arrest and cell death during stress could be interpreted as a mechanism to prevent incorporation of damaged genetic material or transmission of mutation to the subsequent generations. This would raise the question whether Ate1, as a PTM enzyme, has the capacity to affect the outcome of mutagenesis.

**Figure 7:**
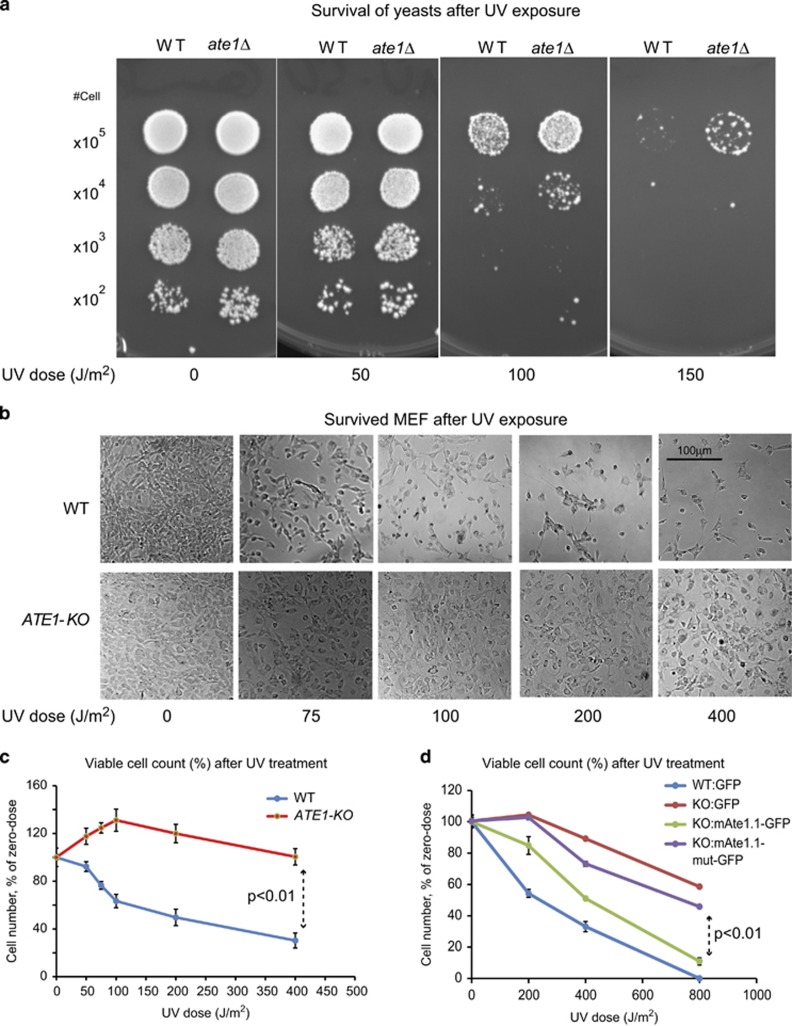
Knockout of *ATE1* increases cell viability upon UV irradiation. (**a**) Representative images of WT and *ate1*Δ yeast grown for 3 days after exposure to different doses of UV irradiation and recovery in the dark. (**b**) Representative images showing WT or *ATE1*-KO MEFs after exposure to increasing doses of UV irradiation and recovery for 24 h. (**c**) Quantification of viable cells at 12 h after UV treatment. Live cells were quantified with cell viability dye Calcein AM and the numbers were normalized to matching samples not irradiated (0 J/m^2^). Error bar represents S.E.M. (*n*=3). (**d**) Comparison of cell viabilities at 12 h after UV treatment for WT or ATE1-KO cells stably expressing either GFP (as transfection and expression control), mAte1.1-GFP or the catalytically impaired mAte1.1-mut-GFP. The number of viable cells was directly counted by cell counter and using Trypan blue to exclude dead cells

To test whether Ate1 affects cellular response to DNA-damaging stress, we subjected cells to ultraviolet (UV) light, a common and natural mutagenic source that generates DNA damage as well as various stressing signals in cells. We found that the *ATE1* deletion in yeast or MEFs significantly increased their resistance to UV light (254 nm UV-C) compared with WT (Figures 7a–c). In addition, the sensitivity to UV in *ATE1*-KO MEFs can be sufficiently reinstalled by the reintroduction of recombinant Ate1 (mAte1.1). This installation effect was nearly abolished when the catalytically impaired mutations (C23-26S) was introduced in Ate1 (Figure 7d).

**Figure 8:**
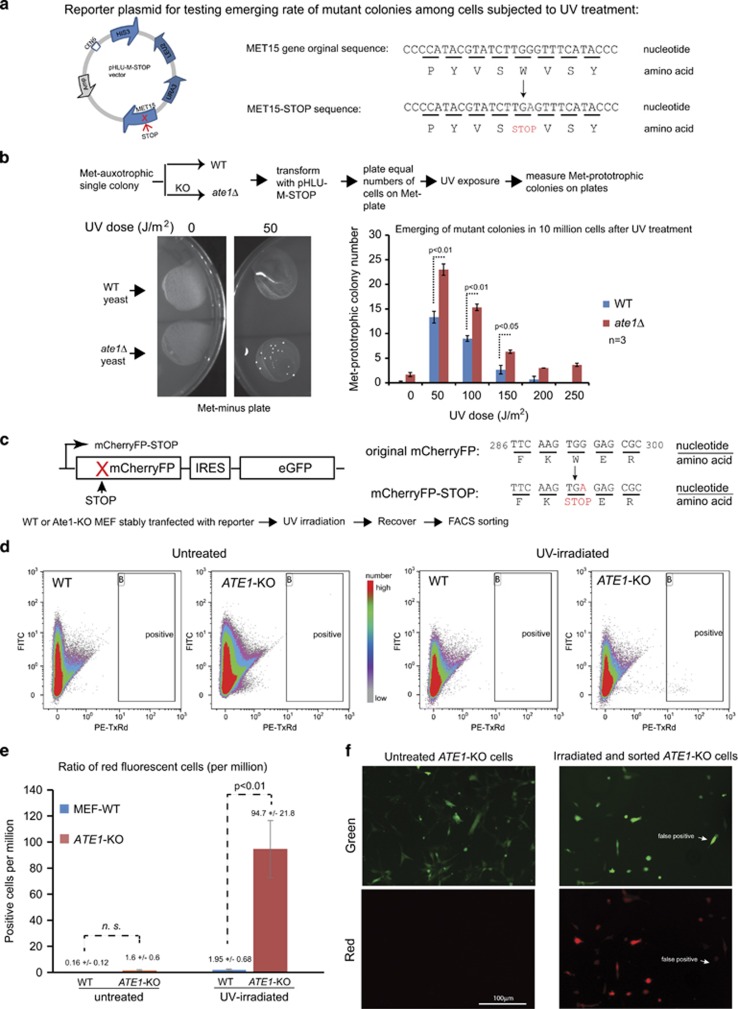
Knockout of *ATE1* increases mutagenesis upon UV irradiation. (**a**) Scheme showing the construction of a mutagenesis reporter plasmid. On the left is the vector map of the pHLUM-stop plasmid, which contains three auxotrophic marker genes: *HIS3*, *LEU2*, *URA3* and a mutated *MET15* gene with a stop codon in the middle of its coding sequence. The scheme on the right shows a portion of the coding sequences and corresponding amino acids in the original *MET15* gene and the mutated *Met15* stop gene, where a TGG codon, coding for tryptophan (W), is converted to a TGA stop codon. (**b**) The top panel shows a flow chart describing the procedure followed to create isogenic pairs of WT and *ate1*Δ yeast and for testing emergence of Met-prototrophic mutant colonies on Met-minus plates starting with the same number of cells for UV irradiation. The bottom left panel has representative images showing the auxotrophic colonies emerged from 20 million yeast (in each spreading) without or with a low dose of UV exposure (50 J/m^2^). The graph on the bottom right is the quantification of the experiment on the left for all tested doses of UV irradiations. Error bar represents S.E.M. (*n*=3, except for the control non-irradiated groups where *n*=6). (**c**) Scheme showing the construction of a mammalian mutagenesis reporter, mCherryFP–STOP–IRES–GFP, which was modified from the pQC-XIG retroviral vector suitable for stable transfection. A STOP codon is inserted in the N-terminal region of the mCherryFP-coding region so that this protein cannot be expressed as full-length, unless an acquired mutation reverts it to a sense codon (revertant). The scheme on the right shows a portion of the coding sequences and corresponding amino acids in the original mCherryFP gene and the mutated mCherryFP–STOP gene, where a TGG codon, coding for tryptophan (W), is converted to a TGA stop codon. (**d**) Representative FACS charts showing the distribution of cell populations by their green and red fluorescence, for WT or *ATE1*-KO MEF, in untreated condition or treated with low-dose UV irradiations that are not expected to lead to significant cell death (two pulses of 20 J/m^2^ irradiations over 48 h, followed by 24 h recovery). The windows marked ‘B' were the gate setting used to quantify and sort red fluorescence-positive cells. (**e**) Quantification of mutated cells showing a red fluorescent signal in FACS in untreated or UV-irradiated cells from four independent repeats (*n*=4). In untreated condition, both WT and *ATE1*-KO cells have negligible numbers of revertants with no significant (NS, *P*>0.05) difference. After UV irradiations, although the WT cells have a moderate increase of revertants (~10 times), the increase in *ATE1*-KO is much larger (~100 times), resulting in a significant difference between the WT and KO cells. Error bar represents S.E.M. As mentioned before, the *P*-value was calculated by Student's *t*-test. (**f**) Representative fluorescent images of *ATE1*-KO MEFs stably expressing the reporter genes, either untreated or treated with UV irradiation and enriched for red fluorescent cells by FACS for culturing of up to 1 week. In untreated cells, no red fluorescence presented in any examined cells. In UV-irradiated and sorted cells, the vast majority of the examined cells have prominent red fluorescence, in addition to the green fluorescence from the internal expression marker GFP on the reporter construct, indicating that they are true revertants. The white arrow in the image points to a false-positive cell, which only has green fluorescence and not red fluorescence. Overall, <5% of false positive was found in the examined cells

To test whether Ate1 is required to prevent DNA mutations in yeast upon UV irradiation, we designed a mutation reporter Met-STOP, in which a tryptophan (TRP/W) residue in *Met15* gene is replaced with an interrupting STOP codon. The product of this engineered gene is not expected to rescue the methionine (Met)-auxotrophy phenotype of BY4741 yeast, unless an acquired mutation reverts the interrupting STOP codon to a sense codon. (Figure 8a). We found that, during non-stressing conditions, WT and *ate1*Δ BY4741 yeast carrying this reporter plasmid similarly generated negligible numbers of colonies on culture plates in the absence of methionine. However, after exposure to UV irradiations, *ate1*Δ yeast produced significantly more colonies acquiring Met-prototrophic phenotype compared with the WT, suggesting that the deletion of Ate1 lead to higher mutation frequencies (Figure 8b). As significant differences between WT and *ate1*Δ yeast were observed in both low and high doses of UV irradiation, it is likely that both the effects of Ate1 in growth arrest and cell death contribute to the mutation suppression.

To validate these observations in mammalian cells, we created a dual-color mutation reporter system mCherryFP–STOP–IRES–GFP, in which an interrupting STOP codon replaces a Trp residue in mCherryFP gene, which is followed by an internal ribosome entry site (IRES) and a eGFP (as the internal control for transcription and expression). As such, this reporter is expected to generate a green fluorescence but not a red fluorescent signal unless a new mutation reverts the interrupting STOP codon in mCherryFP to a sense codon (Figure 8c). To measure the emergence of mutants, the WT or *ATE1*-KO MEFs stably transfected with the reporter, either treated with UV irradiation or not, were analyzed in fluorescence flow cytometry (FACS). In non-stressing conditions, both the WT and *ATE1-KO* cells had negligible numbers of red fluorescent cells. However, when exposed to a low dose of UV irradiation not expected to lead to significant cell death, a significantly higher ratio of red fluorescent cells was detected in the *ATE1-KO* cells compared with the WT cells (Figures 8d and e). To further validate that the red fluorescence detected by FACS was genuine, the positive cells were sorted and subsequently examined by microscopy. We found that the majority of collected cells exhibited both red and green fluorescence, with <5% being false positives having only green fluorescence (Figure 8f). Finally, to confirm the mutation in the reporter gene, we sequenced the corresponding region with genomic DNA prepared from these red-positive cells and identified a reverting mutation in the anticipated location, which was absent in the untreated cells (Supplementary Figure S2).

## Discussion

Although arginylation has been known for more than 50 years, the mechanism underlying the physiological roles of this PTM at the cellular, tissue and organismal levels remain undefined. Our study reveals that Ate1 and Ate1-mediated arginylation are upregulated as part of the general stress response to induce cell growth arrest and cell death, which results in the prevention of DNA mutagenesis under stress. Our data therefore clarify the nature of the involvements of Ate1 and its arginylation activity in stress response, potentially providing a novel explanation for the *in vivo* functions of Ate1 and arginylation in a host of human diseases.

Our data indicate that Ate1 and arginylation have a pro-apoptotic role, which appears to contradict several studies suggesting an anti-apoptotic role.^23, 24, 25, 26, 27, 28^ This discrepancy may be explained by several factors. First, many previous studies focused on individual arginylation substrates. Although those results may still be relevant to the particular pathway studied, they may not be representative of the role of Ate1 or arginylation at the global level. Second, as our data pointed out, Ate1 and arginylation can lead to either growth arrest or cell death, depending on the nature and intensity of the stressor. The growth arrest may occasionally render effects closely resembling anti-apoptotic events. However, our data clearly demonstrate that in the presence of a lethal stressor, Ate1 and arginylation have a pro-apoptotic role.

Our study has unequivocally shown that Ate1 and arginylation are essential for physiological response to a variety of stress conditions including heat, oxidative and osmotic stresses, as well as exposure to heavy metal and radiation. Although there is a certain degree of species variation, overall, a downregulation of Ate1 leads to an increased stress resistance. It is worth pointing out that many of these stressing conditions are known to have causative effects for human diseases such as cancer, cardiovascular disease, aging, developmental abnormalities, injury and inflammation. Interestingly, biochemical and genetic evidence have shown a fluctuation of arginylation activity or disruption of *ATE1* gene being correlated with some of these diseases.^2, 3, 9, 10, 11, 12, 13, 14, 15, 16, 17, 18, 41, 42, 43, 44^ Thus, our results thus have the potential to create new therapeutic interventions based on Ate1/arginylation for these diseases. In particular, our results provide a plausible explanation for the observed but poorly explained correlation between the downregulation of Ate1/arginylation and the increased risk of tumorigenesis, metastasis and radiotherapy resistance.^41, 42, 45, 46^ Tumor microenvironments are often highly stressing due to poor formation of blood vessels or high activity of mitogenic factors. Based on our data, a downregulation of Ate1/arginylation would assist cancer cells to survive the stress, and to accumulate mutations, thereby increasing tumor growth and metastatic risks. These interesting possibilities require further explorations.

Several questions remain open following our study. The complete mechanistic connection between Ate1/arginylation and stress response still awaits elucidation. Also, how Ate1 is being regulated on expression level in normal and transformed cells requires further investigation. In addition, although our data suggest that the effects of Ate1 is largely dependent on its arginylation activity, we cannot dismiss possible involvement of potential regulatory proteins.^37, 47^

Finally, although the actions of other PTM (such as acetylation or phosphorylation) on certain proteins may also affect genetic fidelity,^48, 49^ the global consequence of those modifications have never been demonstrated. Our study is the first example showing that arginylation is a PTM with a unique global effect on mutagenesis.

## Materials and Methods

### Yeast strains

The *S. cerevisiae* strains used in this study are parental WT BY4741 (*MATa his3Δ1 leu2Δ0 met15Δ0 ura3Δ0*) and W303-1A (*MATa leu2-3,112 trp1-1 can1-100 ura3-1 ade2-1 his3-11,15 ybp1-1*), both obtained from Open Biosystems (Lafayette, CO, USA). A strain carrying a null *ate1*Δ:KanMX cassette in the BY4741 background was obtained from Open Biosystems and the cassette was subsequently transferred to the W303-1A background using Ate1-300UP and Ate1-300Down primers. The strain with a GFP fused to the C-terminal end of endogenous Ate1 in the native chromosome locus in BY4741 genetic background was obtained from Open Biosystems. The identity of each KO strain listed above was confirmed with PCR genotyping.

### Culture of yeast

Yeast culture media were prepared as described below:

YPD: 2% glucose, 1% yeast extract and 2% peptone. SD (synthetic defined) medium (per 1000 ml): yeast nitrogen base (1.7 g), ammonium sulfate (5 g), dextrose/galactose/raffinose (20 g), required amino acids (50 mg) and uracil (if required; 50 mg).

For solid media plates, 2% agar was added to the liquid media.

Strains grown in liquid and solid media were incubated at 30 °C unless otherwise indicated.

For most serial dilution growth assays, a single colony of yeast was inoculated in liquid medium and allowed to grow to the log phase before spot plating of serial dilutions as described elsewhere.^50^

### Mammalian cells and media

Immortalized MEFs carrying either WT *ATE1* or KO for *ATE1* (*ATE1*-KO) were a gift from Dr. Anna Kashina (University of Pennsylvania), prepared as described elsewhere.^33^ Immortalized HFFs were a gift from Dr. John Murray (University of Indiana). A human embryonic kidney cell line (HEK 293 T; clone T7) was obtained from ATCC.

Mammalian cells were grown in media containing a 1 : 1 mix of DMEM high glucose and F10 media (Life Technologies, Grand Island, NY, USA, Cat# 11995-065 and 11550-043) with 10% FBS (HyClone, Pittsburgh, PA, USA, Cat# SH30910.03), supplemented with antibiotic-antimycotic (Life Technologies, Cat# 15240-096). The cells were cultured in a 5% CO_2_ incubator at 37 °C, unless otherwise indicated. The MEF cells are continuously split in every 3 days to keep them in active growing stages. Old cells that pass 24 passages were discarded and younger cells were revived from frozen stock. Also, to avoid the interference of contact inhibition, only cells with culture density of <50% were used for any experiments in this study, unless otherwise indicated.

### Molecular cloning and preparation of plasmids

All molecular cloning work was performed with high-fidelity DNA polymerase Herculase (Agilent, Santa Clara, CA, USA). Ligation products were transformed into chemical competent *E. coli* TOP10 (Life Technologies).

PCR reactions were performed in either a Veriti Thermo Cycler (Applied Biosystems, Grand Island, NY, USA) or a T100 Thermo Cycler (Bio-Rad, Hercules, CA, USA). Most primers were ordered from IDT (Coralville, IA, USA) or Sigma (St. Louis, MO, USA).

Primers used for the creation of ATE1 deletion in yeast with the cassette of *ate1*Δ: KanMX

*ATE1*300UP: ATGGTGCTGTGCTTGTAATTG CC

*ATE1*300DOWN: GCTCATCAAAAACTAAGAATAAGAG

The yeast Ate1 overexpression construct was cloned for insertion into the galactose-inducible pYES2 yeast expression vector containing a *GAL1* promoter (Life Technologies, # V825-20) at the *Kpn*1 and *Xba*1 sites using the following primers:

yATE, forward: TACTGGTACCGCCATGTCCGATAGATTCGTTATTTGGGC

yATE, reverse: TAATTCTAGATCACATTTGCTCACTATATAAAATGACGGC

*pGAL*: *ATE1*-GFP cloning: for in-frame fusion of *ATE1* with GFP coding sequence, the stop codon of *ATE1* was removed. Then, a TEV cleavage site linker (ENLYFQGTGGIHRPVAT) was added between Ate1 and GFP, as described before.^37^ The coding sequence for *pGAL*: Ate1-GFP fusion protein was constructed using the following primers for insertion into the pYES2 vector:

yATE-Xho1R: GTATCGTCTAGAGGCGCCCTCGAGCATTTGCTCACTATATAAAATGAC

yATE-GFP, forward: GTATCGCTCGAGGAAAACCTGTATTTTCAGGGAACCGG

yATE-GFP, reverse: GTATCGTCTAGATCACTTGTACAGCTCGTCCATGCCGAGAG

Ate1 point mutations: point mutations of yeast Ate1 for C20,23S were created using the following primers:

yATE-20,23SSF: CAATGAACCTGCCGCAAAGTcTGGGTATTcTCACGGTAATAAGGGGGGC

yATE-20,23SSR: GCCCCCCTTATTACCGTGAgAATACCCAgACTTTGCGGCAGGTTCATTG.

DD-*β*15-GFP, arginylation reporter cloning: the cDNA of the arginylation reporter contains in-frame fusion of the ubiqutin gene on its 5′-region, followed by the coding region of 15 amino acid codons of *β*-actin starting at the third residue (DDIAALVVDNGSGMC), a linker region, and then a coding region for C-terminal GFP. It was cloned in a yeast expression vector, pGPD2 (Addgene, Cambridge, MA, USA, # 43972), at *Eco*R1 and *Xho*1 sites. A plasmid containing the sequence of ubiquitin-DD-*β*-actin was used as a template, as reported.^51^ The following primers were used for this cloning:

Beta-ReporterF: GATAAAGAATTCATGCAAATTTTCGTCAAGAC

Beta-ReporterR: GTCGACCTCGAGTTACTTGTACAGCTCGTCCATG

pHLUM-Met-STOP cloning: a nonsense mutation was created in the *MET15* gene of pHLUM plasmid (Addgene, # 40276) to change nucleotide number 954 from G to A, which converts the Trp318 codon to a STOP codon. For this purpose the following primers were used:

pHLUM-metstop-SnaBF: TCCCCATACGTATCTTGAGTTTCATACCCTGG (containing the mutation site)

pHLUM-metstop-XhoR: GTCTCCCTCGAGCTTGTGAGAGAAAGTAGG

PCR product from the above primers were then digested with restriction enzymes *Sna*BI and *Xho*I, and ligated back to the vector that was digested with the same enzymes.

mCherryFP–STOP cloning: a nonsense mutation was created in the mCherryFP-coding sequence to change nucleotide number 294 from G to A, which converts the Trp98 residue to a STOP codon. For this purpose, the following primers were used:

mCherry-Stop-F: CCCCGAGGGCTTCAAGTGAGAGCGCGTGATGAACTTCG

mCherry-Stop-R: CGAAGTTCATCACGCGCTCTCACTTGAAGCCCTCGGGG

The above primers were used in an overlapping PCR in combination with a pair of cloning primers upstream and downstream of the mCherryFP gene:

mCherry-NotI-F: TAT ATA TGC GGC CGC ATG GTG AGC AAG GGC GAG GAG G

mCherry-TAA-BamHI: ATATGGATCCTTACTTGTACAGCTCGTCCATGCCGCC

The final product was inserted by restriction sites of *Not*I and *Bam*HI before the IRES region in the pQC-XIG vector (Addgene, w497-1).

pBabe-puro vectors containing eGFP or mAte1.1-eGFP were gifts from Dr. Anna Kashina (UPenn). The preparation of those vectors was described elsewhere.^40^ The primers for creating C23,26S mutations on mAte1.1 are:

Forward mutant primer: GCCAGACCTCCTTCCAGaGcGGCTACaGCAAGAACAAGTTGGGCAGTCG

Reverse mutant primer: CGACTGCCCAACTTGTTCTTGCtGTAGCCgCtCTGGAAGGAGGTCTGGC

The below two primers pairs were used with the above primers for overlapping PCR with the original pBabe-mAte1.1-eGFP as template:

Psi-*Spe*I: GCCTGCGTCGGTACTAGTTAG

Ate1-*Eco*RI: GAATGAATTCTTTAGACCCTTC

The final product was digested with *Spe*I and *Eco*RI, and then ligated into the pBabe-mAte1.1-eGFP that was cut with the same enzymes.

### Preparation of protein sample and analysis with SDS-PAGE and western blot

For yeast, the cells were lysed by vortex with glass beads with 1 × Laemmli SDS-loading buffer. The lysate was then heated in a boiling-water bath for 10 min. For mammalian cells, cells were first briefly washed with phosphate-buffered saline (PBS) and then spun down at 2400 × *g* in a desktop centrifuge. The cell pellet was then weighed and lysed with five volumes of PBS and five volumes of 4 × Laemmli SDS-loading buffer and boiled for 10 min, as described.^51^

The protein samples were separated on SDS-PAGE and then transferred to the nitrocellulose membrane for wWestern blot analysis. For GFP or GFP fusion proteins, monoclonal mouse anti-GFP (Roche, Branchburg, NJ, USA, clone 7.1 and 13.1, Cat# 11814460001) was used. For mouse or human Ate1, a monoclonal rat anti-Ate1 (Millipore, Billerica, MA, USA, clone # 6F11, Cat# MABS436) was used. For loading controls, anti-yeast-tubulin (Abcam, Cambridge, MA, USA, # ab6161), anti-yeast-GAPDH (Thermo Fisher Scientific, Grand Island, NY, USA, #MA5-15738), anti-yeast-phosphoglycerate kinase (PGK; Life Technologies #459250) and anti-beta-actin (Sigma, clone AC-15, Cat #A1978) were used. HRP-conjugated secondary antibodies, including anti-mouse/rabbit-HRP (Roche) and anti-rat-HRP (Santa Cruz, Dallas, TX, USA) were used with a chemiluminescence kit (Roche) to visualize the protein bands. The chemiluminescent signals were either documented on film (Denville, Holliston, MA, USA) or by GE Amersham Imager model 600 (GE Healthcare, Pittsburgh, PA, USA). For protein bands documented on film, they were scanned by an Epson Perfection 2400 photo/film scanner (Long Beach, CA, USA) with at least 1200 d.p.i. resolution and then analyzed using Image J (NIH, Bethesda, MD, USA). For protein bands documented by the GE imager, an ImageQuant TL software pack (v8.1) (GE Healthcare, Pittsburgh, PA, USA) and its 1D gel analysis module was used. In addition, fluorophore-conjugated secondary antibodies (Li-COR, Lincoln, NE, USA), including anti-mouse and anti-rat, were used with a LICOR Odyssey imager to visualize protein bands through fluorescence.

### Production of custom antibody against RDD peptide (anti-RDD)

Rabbit polyclonal anti-RDD was ordered from GenScript (Piscataway, NJ, USA) by custom antibody production protocol optimized for recognition of PTMs. In brief, a custom synthesized peptide with the sequence of RDDIAALVVDC (Genscript) was conjugated through the C-terminal cysteine to a carrier protein, keyhole limpet hemocyanin, to increase the presentation of the N terminus of the peptide. The conjugated protein was used as the immunogen for repeated immunization in rabbits. The collected antisera was cross-absorbed by another synthetic peptide with the sequence of DDIAALVVDC (Genscript). The resulted antisera (anti-RDD) was confirmed by immunblots to have minimal cross-reactivity to the peptide DDIAALVVDC, or the N-terminally acetylated peptide Ac-DDIAALVVDC (a gift from Dr. Anna Kashina in the University of Pennsylvania).

### Microscopy

Optical and fluorescent imaging of cells were performed on a Zeiss Observer (Jena, Germany) equipped with a series of objectives and Zen Pro software (Carl Zeiss Microscopy, Jena, Germany).

### Flow cytometry (FACS)

FACS analysis or sorting was performed on a BD Canto-II flow cytometer (BD Biosciences, San Jose, CA, USA) at the core facility of the Sylvester Comprehensive Cancer Center at the University of Miami.

### shRNA-mediated knockdown of Ate1

p.LKO lentiviral vectors containing shRNA targeting GFP, mouse Ate1 and human Ate1 were obtained from the Mission shRNA catalog (Sigma; clone NM_007041.1-520s1c1 for human; clone NM_013799.2-1507s1c1 for mouse). These vectors were packaged using VSV.g and Δ8.2 lentivirus packaging vectors by co-transfecting HEK293T cells with the aid of transfection kit Lipofectamin 2000 (Life Technologies). The viral supernatant was collected at 24, 48 and 72 h, and filtered through a 0.45 *μ*M Syringe filter (Olympus, Genesee Scientific, San Diego, CA, USA, #25-246). The supernatant was then used to transduce MEF and HFF cells, aided by 10 *μ*g/ml polybrene. After transduction, 5 *μ*g/ml puromycin was used to select transduced fibroblast cells for 5–10 days. Once the selected line was stabilized, a western blot was run on a cell lysate to examine changes in Ate1 levels.

### Preparation of stably transfected mammalian cells

Retroviral vector pQC-XIG, and the corresponding vectors for viral core enzymes and surface packaging, GAG-Pol and VSV.g, was obtained from Addgene. Another retroviral vector pBabe-puro containing eGFP or mAte1.1-GFP is gift from Dr. Anna Kashina (UPenn). For virus production, these vectors were co-transfected into HEK293T cells with transfection kit Lipofectamin 2000 (Life Technologies). The viral supernatant was collected at 24, 48 and 72 h, and filtered through a 0.45 *μ*M Syringe filter (Olympus, #25-246). The supernatant was then used to transduce MEF, aided by 10 *μ*g/ml polybrene. The stably transfected cells were then enriched by fluorescent sorting by the GFP signal.

### Stress tests and radiation treatment of mammalian cells

To evaluate the survival of MEFs and HFFs to stress and radiation, 10^4^ cells were plated in each well of a 94-well black-wall plate, with four replicates per dose. The cells were allowed to attach for 4–6 h. For the H_2_O_2_ treatment, H_2_O_2_ (Sigma, Cat# 323381) was added to the cells for 12 h or indicated durations. The cells were then washed with PBS, and cell viability was assessed via Calcein AM staining. For the UV irradiation assay, the culture medium was removed immediately before the irradiation and immediately added back afterward. The cells were then allowed to recover for 24 h at 37 °C, in a CO_2_ incubator before examination or the next treatment. The cell viability was either assessed via staining of live-cell dye Calcein AM or by direct counting in an automated cell counter TC-20 (Bio-Rad) with the aid of cell viability dye Trypan blue.

### Stress treatments of yeast cells

Plate-based yeast stress survival (serial dilution) for NaCl, CdCl_2_ and high temperature was performed on YPD plates. H_2_O_2_ treatment was carried out by overlaying H_2_O_2_ on SD plates.

### Evaluation of yeast cell viability by colony-forming assay

#### *H_2_O_2_ treatment and cell survival*

Cells in log phase growth were collected and suspended (0.2 OD_600_) in SD medium containing various concentration of H_2_O_2_. The treatments were carried out for 3 h at 30 °C with mechanical shaking. Cells were pelleted and washed with water and equal numbers of cells were plated on SD medium plates (containing no H_2_O_2_). Plates were kept at 30 °C and colonies were counted after 5 days. CFUs with 0 mM H_2_O_2_ was set as 100% for normalization separately for each type of yeast.

#### *CdCl_2_ treatment and cell survival*

Cells in log phase growth were collected and suspended (0.2 OD_600_) in SD medium containing CdCl_2_ as indicated in the main text, cultured at 30 °C with mechanical shaking. At given time points, samples of yeast were withdrawn for measurement of OD and/or colony-forming assay. For colony-forming assay, cells were pelleted and washed with water and equal numbers of cells were plated on SD medium plates (containing no CdCl_2_). Plates were kept at 30 °C and colonies were counted after 5 days. CFUs of yeast before the application of CdCl_2_ (time 0) was set as 100% for normalization separately for each type of yeast.

#### *Ate1 overexpression and cell surviva*l

*ate1*Δcells harboring control plasmid or pYES2-*pGAL1*: *ATE1* constructs with the URA3 selection gene marker were initially grown in glucose medium to reach log phase. Cells were then collected, washed and transferred to 2% raffinose medium and grown for 20 h at 30 °C with mechanical shaking to deplete all glucose (which suppresses the *pGAL1* promoter). Cells were then collected, washed and transferred to galactose medium, and grown for various times at 30 °C with mechanical shaking for the induction of protein expression driven by the *pGAL1* promoter. Equal numbers of cells harboring control plasmid or pYES2-*pGAL1*: *ATE1* were plated on Ura-minus, glucose-containing SD medium plates to terminate the galactose induction and measure colony formation abilities of yeast carrying the plasmid. Plates were kept at 30 °C and colonies were counted after 5 days.

### *In vitro* arginylation assay

For *in vitro* arginylation with yeast extract, exponential growth-phase cells of WT BY4741strain yeast (either transfected or not) were used. Equal number of cells treated and not treated with test conditions were lysed in arginylation reaction buffer (50 mM HEPES, pH 7.5, 25 mM KCl, 15 mM MgCl_2_, 2.5 mM ATP, arginine 0.2 mM, PMSF 0.1 M and yeast protease inhibitor cocktail (final dilution 100 × ; Sigma-Aldrich).^37^ The *ate1*Δ yeast expressing DD-*β*15-GFP were also lysed in arginylation buffer. The WT cell lysate was mixed with equal volume of lysate of *ate1*Δ cells expressing the DD-*β*15-GFP to start the arginylation reaction. The reaction was carried out for 10 min at 37 °C. The reaction was stopped by the addition of 1/3 volume of 4 × SDS sample buffer and boiling.

For *in vitro* arginylation with extract of mammalian cells (transfected or not), exponential growth-phase cells of MEFs were collected from culture plate, washed by dPBS and then weighed on scale. The cell pellets were lysed with 2 × volume of a modified reaction buffer (50 mM TRIS/HCl, 32 mM Na_3_PO_4_, pH 7.4, 5 mM MgCl_2_, 1 mM EDTA, 2.5 mM ATP, 0.2 mM arginine and 0.2% NP-40). As an arginylation reporter, recombinant protein DD-*β*15-GFP was expressed in *ate1*Δ yeast and then purified by GFP-TRAPS nanobody conjugated to magnetic beads (Bulldog Bio, Portsmouth, NH, USA) and was shown to be >95% pure by Coomassie-blue staining in SDS-PAGE. This protein was added to the cell lysate as the substrate for arginylation. The reaction was allowed to proceed at RT for 45 min, and terminated by addition of equal volume of 4 × SDS sample buffer and incubation in boiling-water bath for 10 min.

### Evaluation of mammalian cell viability and counting of viable cells by Calcein AM staining

Calcein AM (Life Technologies, Cat C1430) was used at a working concentration of 1 *μ*g/ml in PBS to assess cell viability. Cells were incubated with Calcein AM for 30 min, and fluorescence was measured on a Fluorostar Omega plate reader (BMG Labtech Inc., Cary, NC, USA).

### Direct counting of cell numbers

For cell number counting for yeast or mammalian cells, a TC-20 automated cell counter (Bio-Rad) was used. A viability dye Trypan blue, which only stains dead cells, was also used to facilitate the counting of mammalian cells to differentiate live and dead cells.

### Evaluation of mammalian cell proliferation activity by EdU

Actively growing WT and *ATE1*-KO MEF cells were seeded at 10% density and cultured for up to 18 h with final cell density of <50% confluence. Click-iT EdU Alexa Fluor-594 Imaging kit (ThermoFisher Scientific, catalog number C10339) was used. The cells were pulse labeled with EdU for 45 min at 37 °C in 5% CO_2_ incubator, and then then washed with dPBS and fixed with 3.7% formaldehyde. The cells were then permeabilized with 0.5% Triton X-100 and incubated with the fluorescent labeling reaction cocktail supplied in the Click-iT EdU Imaging kit. Hoechst 33342, also supplied with the kit, was used for DNA staining for identification of cell nuclei as well as their morphologies. Only viable cells with normal nuclear morphologies and with no sign of apoptotic bodies were included in the final quantifications.

### TUNEL assay for apoptosis

Exponential growth-phase cells (WT and Ate1Δ) were collected and suspended (0.2 OD_600_) in SD medium containing 0, 0.1, 0.5 or 2 mM H_2_O_2_. The treatments were carried out for 3 h at 30 °C with mechanical shaking. DNA strand breaks were examined by TUNEL with the In Situ Cell Death Detection kit, fluorescein (Roche Molecular Biochemicals, Branchburg, NJ, USA, # 11684795910), as described earlier. Yeast cells were fixed with 3.7% (vol/vol) formaldehyde for 30 min at RT, washed three times with PBS and cell walls were digested with Lyticase at 2 U per OD cells in 250 *μ*l cell suspension (Sigma-Aldrich, St. Louis, MO, USA, # L2524-10KU) at 37 °C for 1 h. Ten microliters of the cell suspension was applied to a microscope slide and allowed to dry for 30 min at 37 °C. The slides were rinsed with PBS, incubated in permeabilization solution (0.1% (vol/vol) Triton X-100 and 0.1% (wt/wt) sodium citrate) for 2 min on ice, and rinsed twice with PBS. Slides were subsequently incubated with 10 *μ*l of TUNEL reaction mixture, containing terminal deoxynucleotidyl transferase and fluorescein isothiocyanate dUTP, for 60 min at 37 °C. Finally, the slides were rinsed three times with PBS, and a coverslip was mounted. Observations were carried out using Zeiss Observer equipped a series of objectives and the Zen Pro software.

### Annexin-V staining assay for apoptosis

A total of 3x10^5^ MEF cells were seeded onto 60 mM culture-treated dishes and allowed to adhere for 24 h. Following indicated treatments, cells were trypsinized, washed with dPBS and pelleted. Annexin-V and propidium iodide staining was accomplished using the Annexin-V FLUOS kit (Roche, 11858777001). Cells were resuspended in 100ul PBS with 1 : 100 Annexin-V-FITC and 1 : 100 propidium iodide for 15 min at RT. Analysis of staining was performed on a BD Canto-II flow cytometer, using the FITC and propidium iodide channels.

### UV irradiation

UV irradiations of yeast cells or mammalian cells was performed in a 254 nm UV-C irradiation oven (CL-1000S UV crosslinker from UVP, Upland, CA, USA) in a dark room.

### Met-auxotrophy/prototrophy phenotype conversion assay of yeast

WT and Ate1Δ yeast (BY4741 strain) were transformed with construct pHLUM-MET15-STOP (nonsense-trp318STOP). Yeast transformants were selected on SD medium containing methionine but not uracil. Transformants were grown in SD+methionine−uracil medium to log phase. Cultures were collected and washed three times with water and 20 million cells (in 100 *μ*l) were spotted on SD plates (without methionine or Uracil). After any excessive liquid was absorbed by the plate, these cells were exposed to different doses of UV (ranging from 50 to 300 J/m^2^), and then covered with aluminum foil immediately, all performed in the dark. Plates continued to be kept at RT in the dark to avoid triggering photoreactivation-related DNA repair mechanisms. Examination for the emergence of mutant colony began after three days of UV irradiations with continuous monitoring for up to 1 week.

### UV-induced mutagenesis with dual-color reporter in mammalian cells

MEFs (WT or *ATE1*-KO) were stably transfected with the dual-color mutation reporter mCherryFP–STOP–IRES–GFP. Equal numbers of each type of cells were inoculated on 150 mm-diameter cell culture dish for 4–6 h to allow attachment. The final cell density did not exceed 25% confluency. Immediately before UV irradiations, the culture medium was removed. After UV treatment, the original culture medium was added back to the dish and the cells were allowed to recover for 24 h in CO_2_ incubator at 37 °C before another round of UV irradiation. After two rounds of UV irradiations, the cells were allowed to recover for 24 h, before FACS analysis was performed on viable cells that remained attached to the dish.

### Preparation of genomic DNA for Sanger sequencing

A total of 1–5 million of *ATE1*-KO cells carrying stably transfected mutation reporter mCherryFP–STOP–IRES–GFP, which was either untreated or UV-irradiated and sorted for red fluorescence, were used for genomic DNA extraction with Cyclo-Prep Genomic DNA isolation kit (Amresco, Solon, OH, USA) and proteinase K (New England Biolab, Ipswich, MA, USA). The region containing mCherryFP was amplified with high-fidelity polymerase Herculase (Agilent) in two rounds of nested PCR with four primer sets as described below:

To amplify the regions containing mCherryFP on the backbone of vector,

Forward primer targeting CMV promoter: AGAGCTCGTTTAGTGAACCGTC

Reverse primer targeting IRES: ACATATAGACAAACGCACACCG

PCR products with anticipated size from the first round were used as template for next round with these nested primers:

XhoI-CherryF: TTAACTCGAGATTGATCCGCATGGTG

Cherry-NotIR: TTAAGCGGCCGCCGGAATTTTACTTGTAC

The PCR products from the second round amplification with anticipated size were extracted by gel extraction kit (Qiagen, Valencia, CA, USA) and submitted for Sanger sequencing to Eurofins Genomics (Louisville, KY, USA) and Genewiz (South Plainfield, NJ, USA) for double confirmation of results.

## Abbreviations

Ate1: arginyltransferase 1
CFU: colony-forming cells per OD unit
EdU: 5-ethynyl-2′-deoxyuridine
GFP: green fluorescent protein
HFF: human foreskin fibroblast
KO: knockout
MEF: mouse embryonic fibroblast
mAte1: mammalian Ate1
OD: optical density
RT: room temperature
shRNA: short hairpin RNA
STS: staurosporine
TUNEL: Terminal deoxynucleotidyl transferase dUTP nick end labeling
UV: ultraviolet
WT: wild type
