# Supplementary material for: Posttranslational arginylation enzyme Ate1 affects DNA mutagenesis by regulating stress response
Source: Cell Death Dis. 2016 Sep 29;7(9):e2378–. doi: 10.1038/cddis.2016.284 (PMC5059882; doi:10.1038/cddis.2016.284)
Supplement: Supplementary Information [file cddis2016284x1.docx]

**SUPPLEMENTAL MATERIAL**

**Supplemental Figure S1**


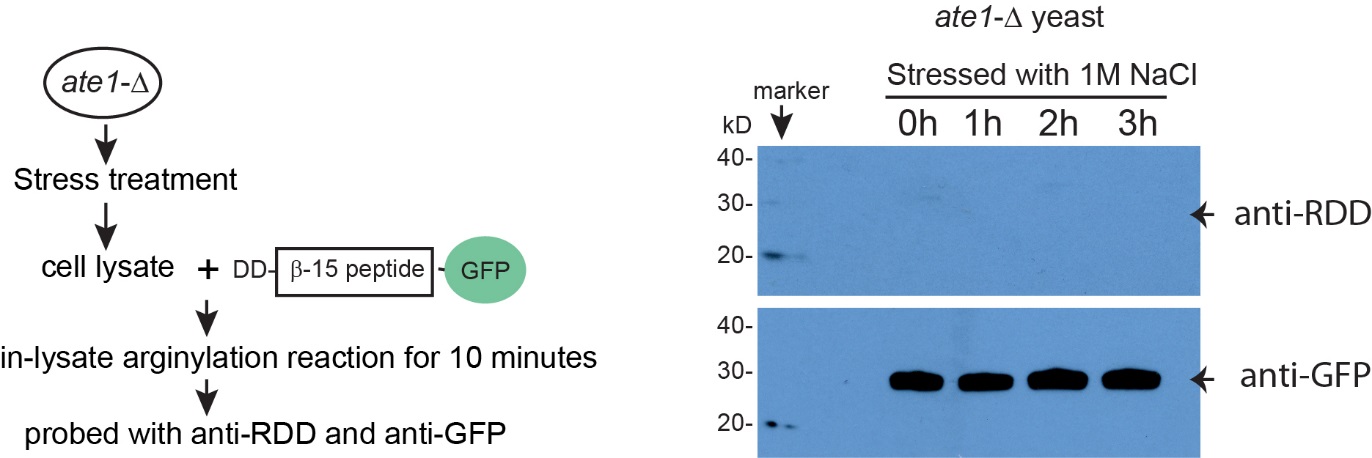


**Supplemental Figure S1. The stress-induced increase of arginylation signal is dependent on the presence of Ate1.**

As a negative control for Fig 3D, left side shows a similar procedure of using in-lysate arginylation assay on *ate1*-Δ yeast exposed to increased duration of 1M NaCl stress. Right side shows a representative immunoblots probed with anti-RDD and anti-GFP, for the detection of arginylated form and the total level of the reporter protein DD-β15-GFP, respectively. The arrows indicated the expected position of the band of DD-β15-GFP on the blots. The protein molecular sizes were indicated with MagicMark XP protein marker (from Invitrogen), which can be visualized by chemifluorescence for positive labeling. As shown in the immunoblots, there was no signal, nor increase of signal, for arginylated DD-β15-GFP in the *ate1*-Δ yeast with stress treatment. In addition, there was no sign of degradation of the substrate. These data suggest that the increase of arginylation signal that we observed in WT yeast shown in Fig. 3C is specifically mediated by Ate1.

**Supplemental Figure S2**


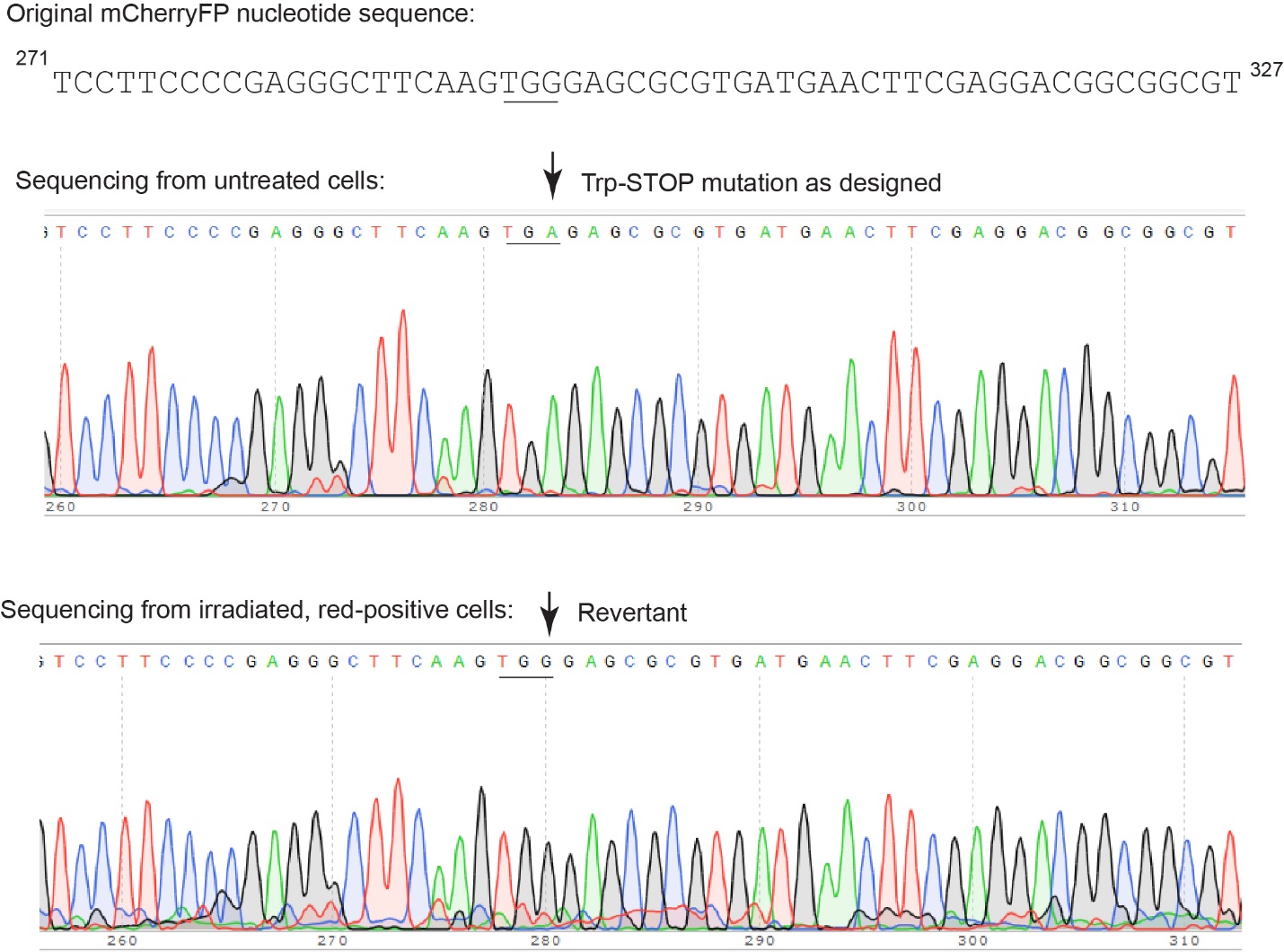


**Supplemental Figure S2. Validation of the reverting mutation by DNA sequencing.**

*ATE1*-KO cells stably with transfected mutation reporter mCherry-STOP were treated with UV-irradiation as shown in Fig.8 and the red-fluorescence positive cells were enriched by sorting and then grown for up to two weeks to reach sufficient cell numbers. Genomic DNA were extracted from those red-positive cells, as well as from untreated cells (as a control for sequencing). The DNA was amplied with two rounds of nested-PCR with two sets of primers that are specific for the region containing the mCherryFP sequence. The PCR products with anticipated size were then submitted for Sanger sequencing. On top of the figure, the original sequence of mCherryFP spanning the target site for mutation (underlined) was shown. In the middle is the sequencing result from untreated cells. Arrow points to the Trp (W)-STOP mutation as designed in the mCherry-STOP reporter. At the bottom is the sequencing result from the expanded culture of the pooled red-positive cells sorted from the UV-irradiated *ATE1*-KO cells carrying the mCherry-STOP vector. Arrows point to the sites of reverting mutation. It is worth noting that, based on the Sanger sequencing result, the detected inversion event is nearly exclusively A -> G, a purine-transition which reverts the STOP codon back to the orginal Trp codon. This could be due to the fact that a transition between purine, rather than a transversion between purine and pyramidine, is more common in genomic mutations[[1](#_ENREF_1)]. Alternatively, it is possible that the Trp residue in this location is essential for the fluorescence of mCherryFP, so that only such a revertant can be detected and collected by FACS.

Reference in Supplemental Material

1. Ebersberger, I., et al., *Genomewide comparison of DNA sequences between humans and chimpanzees.* Am J Hum Genet, 2002. **70**(6): p. 1490-7.
